# XLMR in MRX families 29, 32, 33 and 38 results from the dup24 mutation in the ARX (Aristaless related homeobox) gene

**DOI:** 10.1186/1471-2350-6-16

**Published:** 2005-04-25

**Authors:** Monica L Stepp, A Lauren Cason, Merran Finnis, Marie Mangelsdorf, Elke Holinski-Feder, David Macgregor, Andrée MacMillan, Jeanette JA Holden, Jozef Gecz, Roger E Stevenson, Charles E Schwartz

**Affiliations:** 1 J.C. Self Research Institute, Genetic Center, Greenwood, S.C., USA; 2 Department of Genetic Medicine, Women's and Children's Hospital, Adelaide, Australia; 3 Department of Pediatrics, The University of Adelaide, Adelaide, Australia Ottawa Health Research Institute, Ottawa, Ontario, Canada; 4 Medizinisch Genetisches Zentrum, Bayerstrasse 53, D-80335, Munchen, Munich, Germany; 5 Provincial Medical Genetics Program, St. John, Newfoundland, Canada; 6 Provincial Medical Genetics Program, Health Science Centre, St. John, Newfoundland, Canada; 7 Department Psychiatry & Physiology, Queen's University, Kingston, Ontario, Canada

## Abstract

**Background:**

X-linked mental retardation (XLMR) is the leading cause of mental retardation in males. Mutations in the ARX gene in Xp22.1 have been found in numerous families with both nonsyndromic and syndromic XLMR. The most frequent mutation in this gene is a 24 bp duplication in exon 2. Based on this fact, a panel of XLMR families linked to Xp22 was tested for this particular ARX mutation.

**Methods:**

Genomic DNA from XLMR families linked to Xp22.1 was amplified for exon 2 in ARX using a Cy5 labeled primer pair. The resulting amplicons were sized using the ALFexpress automated sequencer.

**Results:**

A panel of 11 families with X-linked mental retardation was screened for the ARX 24dup mutation. Four nonsyndromic XLMR families – MRX29, MRX32, MRX33 and MRX38 – were found to have this particular gene mutation.

**Conclusion:**

We have identified 4 additional XLMR families with the ARX dup24 mutation from a panel of 11 XLMR families linked to Xp22.1. This finding makes the ARX dup24 mutation the most common mutation in nonsyndromic XLMR families linked to Xp22.1. As this mutation can be readily tested for using an automated sequencer, screening should be considered for any male with nonsyndromic MR of unknown etiology.

## Background

The causes of mental retardation (MR) are exceptionally heterogeneous. More than 50 genes found on the human X chromosome have been reported to cause MR with another 150 loci being associated with linked syndromes, nonsyndromic families and unlinked syndromes [1,2]. The aristaless-related homeobox gene, ARX, maps to Xp22.1-p21.3, encompasses a genomic region of approximately 12.5 kb and is composed of 5 encoding exons [3]. The most frequent mutation reported in ARX is an in-frame 24 bp duplication in exon 2, which causes an expansion of the polyalanine tract at amino acid positions 144–155, from 12 to 20 alanines. This duplication has been reported in a Norwegian family with West Syndrome [3,4] as well as four published nonsyndromic XLMR families, MRX36 [5], MRX43, MRX54 and MRX76 [6]. We have recently identified the same 24 bp duplication in 4 other nonsyndromic XLMR families linked to Xp22.1: MRX29 [7], MRX32 [8], MRX33 [9], and MRX38 [10].

## Methods

Genomic DNA from members of eleven XLMR families linked to Xp22.1, a West Syndrome family with a 21 bp insertion [3], two positive controls for the 24 bp duplication (dup24) [3], and a negative control was amplified by PCR using locus specific primer pairs designed for exon 2 of ARX. The Cy5 labeled forward primer sequence, ARXex2P1, was 5'ACG CCT GGG CCT AGG CAC TG 3' and the reverse primer sequence, ARXex2P1, was 5' CTC GGT GCC GGT GCC ACC AC 3'. These primers flank both the (GCG)_10+7 _and dup24 mutations. The size of the normal control PCR product is 584 bp, while the (GCG)_10+7 _mutation gives a 605 bp product and the dup24 mutation a 608 bp product. The parameters of the reaction consisted of an initial denaturation at 95°C followed by 30 cycles consisting of 95°C for 30 seconds, 62°C for 30 seconds, and 72°C for 30 seconds and completing with a final extension at 72°C for 5 minutes. PCR amplification was verified using a 1.5 % agarose gel, run at 120 volts, and stained with ethidium bromide. PCR products were then analyzed using the Amersham Biosciences ALFexpress1.

## Results

**Figure 1:**
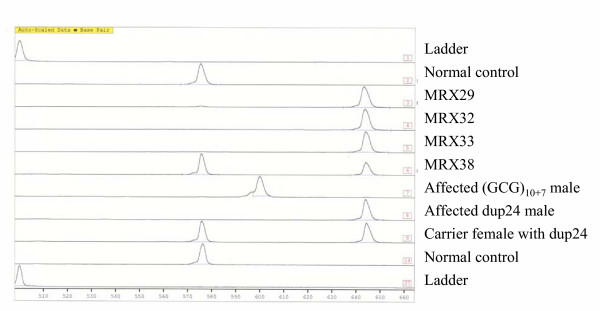
Chromatograph of samples with ARX 24dup mutation. Lane 17 is the 50–500 bp ladder lanes. Lane 14 is a normal control. Lane 3 is an affected male from MRX29. Lane 4 is an affected male from MRX32. Lane 5 is an affected male from MRX33. Lane 6 is a female carrier from MRX38. Lane 7 is a male with (GCG)10+7 ARX mutation. Lane 8 is an affected male with a known dup24 mutation and Lane 9 is a female carrier with the dup24 mutation.

The analysis revealed an altered pattern in a female carrier of MRX38 and one affected male from each of three other families: MRX29, MRX32, MRX33 (Figure 1). The pattern was identical to that observed for two samples known to have the dup24 mutation in exon 2 of ARX and clearly distinct from the 21 bp insertion in exon 2 that is present in the patient with West Syndrome (Figure 1). DNA sequencing of the PCR products confirmed the presence of the dup24 mutation. It is important to note the discrepancy in the size of the dup24 mutation and the sizing standard in lanes 1 and 4 (Figure 1). We hypothesize that the slower migration pattern observed for the dup24 mutation (~645 bp instead of the expected 608 bp) is due to its secondary (hairpin) structure.

## Discussion

We have identified the dup24 mutation in exon 2 of ARX in four additional nonsyndromic XLMR families (MRX29, 32, 33, and 38) linked to Xp22.1. This same alteration has been observed in four other published nonsyndromic families, MRX36 [5], MRX43, MRX56 and MRX76 [6] linked to Xp22.1. As a result, this single mutation accounts for 8/11 (73%) of MRX families linked to Xp22.1 [11]. This 24 bp duplication is not restricted to nonsyndromic XLMR families. A family with West syndrome linked to Xp22.1 had this alteration [4], as well as 2 families with Partington syndrome, also linked to Xp22.1 [3]. This association of nonsyndromic and syndromic XLMR with the 24 bp duplication has also been noted by Kato et al [12].

The dup24 also accounts for 2/10 (20%) syndromic XLMR conditions linked to Xp22.1 [13,14]. This is quite striking and noteworthy since for most XLMR genes described thus far, mutations are found in less than 20% of families linked to any particular region [15]. Overall, it appears that when all mutations in ARX are considered, alterations in the gene accounts for 4/58 (7%) syndromic and 8/24 (25%) nonsyndromic XLMR conditions with known gene defects. Again, this is uncharacteristic of other XLMR genes, except for ATRX/XNP (mutations in 6 syndromes) or PQBP1 (mutations 5 syndromes) [14].

This being said, there is a "disconnect" when screening for ARX mutations is expanded to the general male MR population. Bienvenu et al. [6] were only able to identify two mutations in a panel of 148 XLMR families and only one dup24 mutation in 40 sporadic males with MR. Additionally, Gronskov et al. [16] identified only one dup24 mutation out of 682 patients. Other studies experienced a similar failure to find this mutation in 577 males with non-fragile X MR (Stepp and Schwartz, unpublished) and a cohort of 188 male patients [15]. This translates to a positive screen rate of 2/1501 male patients with MR, significantly different from the 6.6% rate observed in proven XLMR families [15].

At present, there is no clear explanation for this disparity in the frequency of ARX mutations between families with XLMR linked to Xp22.1 and sporadic cases of males with MR. It may simply mean that the assumption that the 25–30% excess of males with MR is due to genes on the X chromosomes is incorrect. Therefore, the male excess in the MR population might have to be accounted for by other mechanisms including yet to be revealed X-chromosome linked factors.

## Conclusion

In conclusion, we have shown that 4 additional MRX families (MRX29, 32, 33 and 38), mapped to Xp22.1, have the dup24 mutation in exon 2 of the ARX gene. This mutation accounts for about 70% of MRX families linked to Xp22.1. However, it has been difficult to find this same mutation in large cohorts of males with mental retardation. Nonetheless, as the 24dup mutation in ARX can easily be tested using an automated DNA sequencer, screening of males with MR of unknown etiology for this particular mutation should be given consideration.

## Competing interests

The author(s) declare that they have no competing interests.

## Authors' contributions

MLS, ALC, MF and MM carried out the molecular genetics studies. EH-F, DM, AM, JJAH and RES provided clinical material. JG and CES drafted the manuscript. CES conceived the study and participated in its design and coordination. All authors read and approved the final manuscript.

## Pre-publication history

The pre-publication history for this paper can be accessed here:
